# Reactive Arthritis Secondary to Cauda Equina Injury following Spine Fracture: A Case Report

**DOI:** 10.1155/2011/648080

**Published:** 2011-12-22

**Authors:** Xiao Li, Shaoling Wu, Chao Ma

**Affiliations:** Department of Rehabilitation Medicine, Sun Yat-sen Memorial Hospital, Sun Yat-sen University, Guangdong, Guangzhou 510120, China

## Abstract

A 38-year-old man presented with a one-month history of muscle weakness and dysesthesia in the lower extremities, urinary retention, and urinary tract infection after lumbar burst fracture resulted from high fall. During the rehabilitation in our hospital, he had arthritis in both the ankle and knee. However, the patient was treated as gouty arthropathy initially. The arthritis was completely remitted in a few days after the patient was diagnosed as reactive arthritis and started with sulfasalazine therapy and there was no recurrence during 4 months of follow-up. Based on this case, early recognition of reactive arthritis is of major importance to avoid delayed initiation of appropriate treatment in the patients with polyarthritis secondary to neurogenic bladder following cauda equina injury after spine fracture.

## 1. Introduction

Reactive arthritis (ReA) is an inflammatory arthritis that arises after certain types of gastrointestinal or genitourinary infections, representing the classic interplay of host and environment [1]. The classic presentation of ReA is a triad of symptoms, including the urethritis, conjunctivitis, and synovitis. However, the majority of patients do not present with this classic triad [2].

Neurogenic bladder (NB) is often associated with spinal cord diseases or cauda equina injury after spine fracture but may also be caused by brain diseases or peripheral nerve diseases. The urological complications of NB consist of an inability to empty the bladder, as well as urinary tract infections, incontinence, and upper tract deterioration. Urinary tract infection is a common problem among patients with spinal cord injury (SCI) accounting for 50% of nosocomial infections in some international studies [3]. The overall rate of urinary infection in SCI patient is about 2.5 episodes per patient per year [4].

Reviewing literatures, neurogenic bladder in population with spinal cord injury or cauda equina injury has been well recognized, but reactive arthritis resulting from spinal cord injury or cauda equina injury is very uncommon. This is the first report of the clinical course of a patient with reactive arthritis secondary to neurogenic bladder following cauda equina injury in China. The diagnostic and therapeutic approach to the patient and details of its evolution are presented below. 

## 2. Case Report

A 38-year-old man was admitted to our hospital complaining of a one-month history of muscle weakness and dysesthesia in the lower extremities, urinary retention accompanied with urinary tract infection on January 14, 2011. He fell from the second floor of a block of flats on December 2, 2010 and plain radiograph of the thoracolumbar spine revealed a comminuted, burst fracture of  L_1_ and L_2_. Then emergency surgical treatment was performed for removal of intradural space-occupying lesion and decompression of cauda equina. In spite of this, he had the onset of urinary retention, muscle weakness, and dysesthesia in the lower extremities resulting from damage to the cauda equine after operation. During further rehabilitation in community hospital, the urinary tract infection developed. The laboratory investigation showed white blood cell (WBC) in the urinewas higher than the normal level. After treatment with oral antibiotic, the urinary tract infection improved.

He was hospitalized in our department for further treatment and the diagnosis of cauda equine injury with neurogenic bladder was based on laboratory and clinical information below. Upon admission, his body temperature was 36.7°C, pulse rate was regular, 78 beats/min, and blood pressure was 120/78 mmHg. The neurologic examination was compatible with a cauda equine lesion: there was hypesthesia at left L_1_ and right L_2_ sensory dermatome, hyporeflexia knee and ankle jerks, no anal sphincter contraction, and bulbocavernosus reflex. His white blood count was 6.99/*μ*L, hemoglobin 126 g/L, and platelets 250/*μ*L. Differential count showed 69.5% neutrophils, 20.7% lymphocytes, 6.7% monocytes, 2.7% eosinophils, and 0.4% basophils. White blood cell count in the urine was 1169/*μ*L (normal range 0~12/*μ*L). On X-ray of the thorax, knee, feet, and ankles, no abnormalities were observed. Ultrasonography revealed that there was residual urine in the bladder and the volume was higher than normal (>150 mL). Because of the neurogenic bladder, he was subjected to a series of treatments including intermittent urethral catheterization and different bladder management methods (Valsalva, Crede, and reflex voiding). A microscopic urine analysis revealed WBC, and bacterial colony counts were still higher than the normal level. It suggested that the urinary infection were not completely curable. And then the patient was treated with sensitive antibiotic (levofloxacin, i.v. infusion) and bladder wash-out method in 1/5000 furacin. Urinalysis revealed that the WBC count in the urine was at the normal level 2 weeks later.

On February 5, 2011, the patient reported joint pain of his left ankle. Physical examination revealed swelling and tenderness of the involved joint. Laboratory testing showed erythrocyte sedimentation rate (ESR) 50 mm/h (normal range < 15 mm/h), C-reactive protein (CRP) 46.3 mg/L (normal range < 3 mg/L), serum uric acid 527 *μ*mol/L (normal range 120~452 *μ*mol/L), and serum amyloid A (SAA) 8.5 mg/L (normal range < 6.8 mg/L). Rheumatoid factor (RF) and anti-nuclear antibody (ANA) were negative. The examination of liver and renal function was normal. X-ray of the left ankle joint showed periarticular soft tissue swelling, and there were no erosive changes. Ultrasonography revealed tibialis posticus tendonitis on the left. There was no embolization in the vessels of left lower limb according to color doppler ultrasonography.

On March 2, 2011, the patient complained of swelling and joint pain involving his left knee and subsequently developed restricted range of motion next morning. On March 5, 2011, he presented a relapse of painful swelling of left ankle. Physical examination revealed that floating patella test was positive. Laboratory data was as follows: ESR 50 mm/h, CRP 26.3 mg/L, serum uric acid 525 *μ*mol/L, SAA 13.1 mg/L, and anti-deoxyribonuclease B 382 U/mL (normal range < 200). Serological tests for RF, ANA, antineutrophil cytoplasmic antibody, complement factor C3, C4, antistreptolysin-O antibody, syphilis, gonorrhea, hepatitis B, HIV, and HLA-B27 were negative. The serum IgG, IgA, and IgM were 9.41, 1.77, and 0.71 g/L, and IgE was 8 IU/mL, respectively (all normal range). Aspiration from the left knee yielded large amounts of clear, straw-coloured fluid of low viscosity with leucocytes 1850/dl and no crystals. Standard cultures of all obtained synovial fluid samples were negative. During hospitalization the patient had no fever, chills, coughing, sputum, diarrhea, conjunctivitis, and mucous membrane lesion.

The patient was initially identified as gouty arthropathy and therapy with allopurinol and celecoxib was started. Nevertheless, it did not offer any meaningful benefit. Consulting the rheumatologist, the patient was subsequently treated with colchicines, methylprednisolone, and celecoxib for one month. Despite the drugs above administration, the pain and swelling persisted. Reviewing the clinical history of this case, a diagnosis of reactive arthritis was confirmed and colchicine was replaced by sulfasalazine [5, 6] to which he responded. The arthritis was completely remitted in a few days after that and there was no recurrence during 4 months of follow-up.

## 3. Discussion

Reactive arthritis can be defined as a sterile joint inflammation that develops after a distant infection [7]. The classic presentation of ReA is characterized by an asymmetric arthritis usually in the lower limbs associated with urethritis, conjunctivitis, and occurrence of other articular or extra-articular manifestations [8, 9]. There are few reports about ReA secondary to neurogenic bladder after spine fracture. By studying the PubMed/MEDLINE database, Mukul P. Agarwal reported a case of a 32-year-old male who presented with myelitis in association with reactive arthritis [10].

The symptoms of neurogenic bladder can be divided into detrusor overactivity which can occur in the central nervous system disorders and disturbed voiding due to poor relaxation of urethral sphincter or detrusor weakness [11]. Recurrent urinary tract infection is one of the commonest complications of neurogenic bladder because of incomplete bladder emptying and the use of catheters that can result in the introduction of bacteria into the bladder [12, 13]. Our patient had a cauda equina injury caused by fracture of lumbar vertebra (L_1_, L_2_) that led to disturbed voiding.

Although pharmacotherapy and intermittent catheterization were used in our patient before he was admitted into our hospital, he presented with disturbed voiding and increased residual urine volume (>150 mL) after operation. The patient was further treated with sensitive antibiotics according to urine culture [14]. At the same time, he learned about clean intermittent catheterization and bladder function training, such as Crede maneuver and Valsalva maneuver [15, 16]. The urinary tract infection was controlled completely after one-month therapy, and ultrasonography showed that the residual urine volume was below 50 mL in the meantime. During the rehabilitation in our hospital, he had arthritis in both the ankle and knee. Due to loss of early recognition and comprehensive evaluation of the disease, the patient was treated as gouty arthropathy initially and there was no great relief of symptoms. Our case demonstrated the prompt and sustained ameliorative effect of sulfasalazine on symptoms caused by reactive arthritis.

In conclusion, we report on a patient with the rare association of reactive arthritis, neurogenic bladder, cauda equina injury, and spine fracture. Neurogenic bladder is a common complication of cauda equina injury, and there will be recurrent urinary tract infections when the NB is not controlled well. As a trigger, the urinary tract infection lead to reactive arthritis [17–19]. So reactive arthritis must be kept in mind in the patients with polyarthritis secondary to urinary tract infection following neurogenic bladder. Correct identification of reactive arthritis may avoid delayed initiation of appropriate treatment.
